# Lutetium-177-PSMA-I&T as metastases directed therapy in oligometastatic hormone sensitive prostate cancer, a randomized controlled trial

**DOI:** 10.1186/s12885-020-07386-z

**Published:** 2020-09-14

**Authors:** Bastiaan M. Privé, Marcel J. R. Janssen, Inge M. van Oort, Constantijn H. J. Muselaers, Marianne A. Jonker, Michel de Groot, Niven Mehra, J. Fred Verzijlbergen, Tom W. J. Scheenen, Patrik Zámecnik, Jelle O. Barentsz, Martin Gotthardt, Walter Noordzij, Wouter V. Vogel, Andries M. Bergman, Henk G. van der Poel, André N. Vis, Daniela E. Oprea-Lager, Winald R. Gerritsen, J. Alfred Witjes, James Nagarajah

**Affiliations:** 1 Department of Radiology and Nuclear Medicine, Radboudumc, Geert Grooteplein Zuid 10, 6525 GA Nijmegen, The Netherlands; 2 Department of Urology, Radboudumc, Nijmegen, The Netherlands; 3 Department of Health Evidence, Radboudumc, Nijmegen, The Netherlands; 4 Department of Medical Oncology, Radboudumc, Nijmegen, The Netherlands; 5 Department of Radiology and Nuclear Medicine, University Medical Center Groningen, Groningen, The Netherlands; 6 Department of Radiology and Nuclear Medicine, NKI Antoni van Leeuwenhoek Hospital, Amsterdam, The Netherlands; 7 Department of Radiation Oncology, NKI Antoni van Leeuwenhoek Hospital, Amsterdam, The Netherlands; 8 Department of Medical Oncology, NKI Antoni van Leeuwenhoek Hospital, Amsterdam, The Netherlands; 9 Department of Urology, NKI Antoni van Leeuwenhoek Hospital, Amsterdam, The Netherlands; 10 Department of Urology, Amsterdam University Medical Center, Amsterdam, The Netherlands; 11 Department of Radiology and Nuclear Medicine, Amsterdam University Medical Center, Amsterdam, The Netherlands

**Keywords:** Hormone sensitive prostate Cancer, Lutetium-177-PSMA, Metastases directed therapy, Oligometastases, Radioligand therapy, Urologic oncology

## Abstract

**Background:**

In recent years, there is increasing evidence showing a beneficial outcome (e.g. progression free survival; PFS) after metastases-directed therapy (MDT) with external beam radiotherapy (EBRT) or targeted surgery for oligometastatic hormone sensitive prostate cancer (oHSPC). However, many patients do not qualify for these treatments due to prior interventions or tumor location. Such oligometastatic patients could benefit from radioligand therapy (RLT) with ^177^Lu-PSMA; a novel tumor targeting therapy for end-stage metastatic castration-resistant prostate cancer (mCRPC). Especially because RLT could be more effective in low volume disease, such as the oligometastatic status, due to high uptake of radioligands in smaller lesions. To test the hypothesis that ^177^Lu-PSMA is an effective treatment in oHSPC to prolong PFS and postpone the need for androgen deprivation therapy (ADT), we initiated a multicenter randomized clinical trial. This is globally, the first prospective study using ^177^Lu-PSMA-I&T in a randomized multicenter setting.

**Methods & design:**

This study compares ^177^Lu-PSMA-I&T MDT to the current standard of care (SOC); deferred ADT. Fifty-eight patients with oHSPC (≤5 metastases on PSMA PET) and high PSMA uptake (SUVmax > 15, partial volume corrected) on ^18^F-PSMA PET after prior surgery and/or EBRT and a PSA doubling time of < 6 months, will be randomized in a 1:1 ratio. The patients randomized to the interventional arm will be eligible for two cycles of 7.4GBq ^177^Lu-PSMA-I&T at a 6-week interval. After both cycles, patients are monitored every 3 weeks (including adverse events, QoL- and xerostomia questionnaires and laboratory testing) at the outpatient clinic. Twenty-four weeks after cycle two an end of study evaluation is planned together with another ^18^F-PSMA PET and (whole body) MRI. Patients in the SOC arm are eligible to receive ^177^Lu-PSMA-I&T after meeting the primary study objective, which is the fraction of patients who show disease progression during the study follow up. A second primary objective is the time to disease progression. Disease progression is defined as a 100% increase in PSA from baseline or clinical progression.

**Discussion:**

This is the first prospective randomized clinical study assessing the therapeutic efficacy and toxicity of ^177^Lu-PSMA-I&T for patients with oHSPC.

**Trial registration:**

Clinicaltrials.gov identifier: NCT04443062.

## Background

Prostate cancer (PC) is the most common non-skin cancer in males [1]. Despite surgery or external beam radiotherapy (EBRT), approximately 20–40% of patients will eventually have a detectable prostate-specific antigen (PSA) and present with disease recurrence [2, 3]. If there are no curative options, patients with a short PSA doubling time (e.g. < 6 months) have a poorer prognosis and early androgen deprivation therapy (ADT) is the treatment of choice [4–6]. While ADT delays disease progression of patients, it is associated with significant side effects and frequently impairs the quality of life [7]. Therefore, there is an increasing interest in treatments to postpone ADT while maintaining good quality of life.

In recent years, metastases directed therapy (MDT) (e.g. targeted surgery or EBRT) attracted much attention to postpone ADT or even with potential cure for selected patients. Particularly, patients with a limited number of metastases (≤5 metastases), so called ‘oligometastatic’ PC, seem to benefit from MDT. Here, EBRT offers an ADT free survival of 14 to 29 months with solely low-grade treatment related side effects [8–12]. Therefore, several clinical trials are currently investigating MDT in this hormone sensitive oligometastatic setting (clinicaltrials.gov: NCT03569241, NCT04075305, NCT02170181, NCT04302454, NCT02192788, NCT02685397, NCT04115007, NCT02264379, NCT02680587, NCT03630666, NCT02274779, NCT03795207, NCT03525288, NCT03784755).

Oligometastases was first described in 1995 by Hellman and Weichselbaum [13]. This disease status became relevant when novel imaging modalities, such as prostate specific membrane antigen positron emission tomography (PSMA-PET), were introduced with better tumor detection rates compared to the conventional scans (e.g. CT or bone scans) [14, 15]. Consequently, all the above-mentioned studies utilizes PET to detect and target the tumor (metastases) [14]. The current favored PET tracers in PC are Gallium-68 (^68^Ga) or Fluor-18 (^18^F) labeled prostate-specific membrane antigen (PSMA) ligands. PSMA is highly overexpressed in > 90% of PC cells and seem to increase with the aggressiveness of the tumor [16, 17]. Several prospective studies have shown that ^68^Ga-PSMA PET has an excellent sensitivity and specificity (> 85 and 98%, respectively) to detect PC [15, 18, 19]. However, PSMA ligands, such as PSMA-617 & PSMA-I&T, can also be labeled with beta emitters like Lutetium-177 (^177^Lu) for radioligand therapy to deliver high local radiation doses to tumors directly [20–22].

^177^Lu is a beta (β^−^) radiation emitter with a maximum energy of 0.50 MeV with maximum penetration depth of 2 mm and a 6.7-days half-life. ^177^Lu labelled PSMA is a promising new therapeutic approach and frequently used in compassionate use programs worldwide [22–26]. To date, only one prospective trial of ^177^Lu-PSMA-617 has been published, showing efficacy in 57% of end-stage PC patients and ^177^Lu-PSMA was generally well tolerated [20]. These observations were recently confirmed at ASCO, with the presentation of the initial results of the TheraP trial comparing ^177^Lu-PSMA-617 to cabazitaxel in mCRPC patients (NCT03392428) [26]. The pivotal trial of ^177^Lu-PSMA-617 in end-stage PC, called the VISION study (clinicaltrial.gov identifier: NCT03511664), is currently being finalized. However, based on the mode of action and in concordance to results from our pilot study (clinicaltrials.gov identifier: NCT03828838), ^177^Lu-PSMA is also highly effective in low volume disease because of high tumor uptake of PSMA targeted radioligands in small lesions, such as oHSPC [27–29]. Moreover, the favorable toxicity profile of ^177^Lu-PSMA seen in our pilot study supports this new treatment in this setting. Hence, we initiated a prospective randomized multicenter study analyzing the efficacy of ^177^Lu-PSMA in oHSPC to postpone disease progression and averting the need for ADT.

## Methods and design

The study protocol was approved by the Medical Review Ethics Committee Arnhem-Nijmegen, The Netherlands and is registered on clinicaltrials.gov (NCT04443062).

**Fig. 1:**
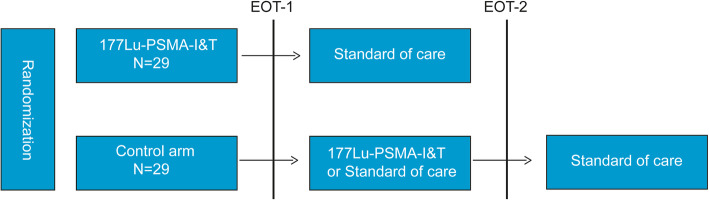
A scheme of the trial with the randomization and study EOT stages. EOT = End of Treatment; PSMA = prostate specific membrane antigen

This is a two-arm randomized open label multicenter phase II study performed in Radboudumc Nijmegen, Netherlands Cancer Institute Antoni van Leeuwenhoek Hospital Amsterdam, Amsterdam University Medical Center and University Medical Center Groningen. This study will compare ^177^Lu-PSMA-I&T MDT in oligometastatic (≤5 metastases on ^18^F-PSMA PET/CT) PC to the current standard of care (SOC), which is watchful waiting till initiation of ADT [6]. This study will include 58 patients with a PSA doubling time of < 6 months, a patient cohort prone to initiate ADT [30]. However, all patients, including the SOC arm, will have access to ^177^Lu-PSMA-I&T (including the follow up schedule), but only if the primary endpoint is reached and disease progression has occurred (EOT 1) and the patients are willing to undergo ^177^Lu-PSMA RLT (Fig. 1). This design enables not only a comparison of ^177^Lu-PSMA-I&T with SOC in a randomized setting but also analyze the efficacy of ^177^Lu-PSMA-I&T in progressing PC patients, which may violate the inclusion criteria of “oligometastatic”. Moreover, with this strategy, we anticipate to prevent drop-offs in the SOC arm what the VISION study frequently encountered [31]. The therapeutic arm patients (and SOC patients after EOT 1) will receive two cycles of 7.4GBq ^177^Lu-PSMA-I&T each. This is less than the current recommended schedule for end-stage PC patients with 4–6 cycles of 7.4GBq ^177^Lu-PSMA each [20, 23]. This schedule is based on the dosimetry results from our pilot study [28]. In the present study, the ligand PSMA-I&T will be used, which was to date not yet investigated in a prospective randomized study world-wide. Nevertheless, ^177^Lu-PSMA-I&T has shown to be efficient in retrospective setting in end-stage PC patients [22].

### Objectives

The primary objective is:

- To study the effect of ^177^Lu-PSMA-I&T in patients with oHSPC, by comparing the fraction of patients that have disease progression and meet EOT 1 criteria within 6 months after cycle two in a group of patients that are treated with ^177^Lu-PSMA-I&T and a group that follows the current SOC (deferred ADT).
- A second primary objective is to compare the two arms for the time to disease progression and meeting EOT 1 criteria.

Secondary Objectives are:

- To evaluate the clinical efficacy of multiple doses ^177^Lu-PSMA-I&T in patients with oHSPC by:
  - The change in PSA after ^177^Lu-PSMA-I&T and proportion of achieving a ≥ 50% decrease in PSA from baseline.
  - The changes in uptake (SUV) of ^18^F-PSMA PET/CT before and 6 months after ^177^Lu-PSMA-I&T.
  - The changes in number and size of (soft) tissue metastases on ^18^F-PSMA PET/CT and (whole body) MRI after ^177^Lu-PSMA-I&T.
- To evaluate the PFS, which is defined as the time from ‘cycle one, day one’ to date of evidence of: clinical progression, PSA progression, or radiographic progression and death from any cause.
  - Clinical progression is defined by the treating physician (e.g. increasing pain from metastases).
  - PSA progression is defined as a ≥ 25% increase in PSA from nadir, with a minimum PSA of > 0,5 μg/l and which is confirmed by a second value ≥3 weeks later (i.e. confirmed rising trend). Within the first 12 weeks after treatment administration PSA increases will be ignored in the absence of other evidence of disease progression due to possible flare phenomenon. If no decline occurs, initial date of ≥25% increase will be recorded [32].
  - Radiographic progression is defined by the amount and size of the lesions. Where applicable Prostate Cancer Working Group 3 (PCWG3) and Response Evaluation Criteria in Solid Tumors (RECIST) v1.1 criteria will be followed
- To assess ADT free survival in patients receiving ^177^Lu-PSMA-I&T. ADT free survival is defined by the date any ADT (e.g. bicalutamide, luteinizing hormone-releasing hormone drugs, enzalutamide, abiraterone, etc.) is started or death related to PC.
- To evaluate the tolerability and toxicity of ^177^Lu-PSMA-I&T defined by NCI Common Terminology Criteria for Adverse Events (CTCAE) v5.0.
- To evaluate the quality of life before and up to 6 months after ^177^Lu-PSMA-I&T using the following questionnaires: EORTC QLQ-C30, QLQ-PR25 and xerostomia inventory.

### Study endpoints

When patients have disease progression and meet EOT 1 criteria during study follow up, the primary study objective can be elucidated. EOT 1 is defined by:

- A 100% increase in PSA from ‘cycle one, day one’ blood draw (BASELINE) during study. Exception: PSA increase in the first 12 weeks after the first treatment injection as was defined by the PCWG3 criteria [32].
- Clinical progression determined by the treating physician (e.g. increasing pain from metastases)

After answering the primary research question (EOT 1), patients randomized to the SOC arm are eligible to receive ^177^Lu-PSMA-I&T, if both the treating physician and the patient agree to continue with the trial. These study results will be analyzed separately for secondary study objectives. Although the cohorts might not be completely similar (e.g. higher tumor volume, no PSA doubling time of < 6 months), we can include intra-individual analyses in a group that has clearly progressing PC. When these SOC patients continue to have progressive PC (defined as EOT 2) despite of ^177^Lu-PSMA-I&T, an end of study visit should be arranged within 4 weeks, but prior to starting ADT. EOT 2 is defined by:

- A 100% increase in PSA from ‘EOT 1, cycle one, day one’ blood draw (NEW BASELINE) during the study follow up. Exception: PSA increase in the first 12 weeks after the first treatment injection as was defined by the PCWG3 criteria [32].
- Clinical progression determined by the treating physician (e.g. increasing pain from metastases)

### Inclusion criteria

In order to participate in this study, a subject must meet all of the following criteria:

- Histological proven adenocarcinoma of the prostate with sufficient archived tumor material. This material has to be archived till study closure.
- Biochemical recurrence (PSA > 1.0 μg/l).
- PSA-doubling time < 6 months. Serum PSA progression is defined as 2 consecutive rising PSA values measured at least 1 week apart. The minimal start value is 0.2 μg/l.
- ^18^F-PSMA-PET/CT positive metastases in bones and/or lymph nodes (N1/M1ab): ≥1, maximally 5 metastases.
- Local treatment for oligometastases with radiotherapy or surgery appears to be no option anymore (due to prior treatment or the location of the metastatic lesions or if the patient refuses these treatments).
- No prior hormonal therapy (including any androgen directed treatment such as finasteride, dutasteride, bicalutamide, apalutamide, abiraterone or enzalutamide) or taxane based chemotherapy (docetaxel or cabazitaxel); testosterone > 1.7 nmol/l.

*Exception: local PC treated with local radiotherapy plus adjuvant ADT; these patients need to be stopped with ADT at least 6 months.*

- A detectable lesion on the ^18^F-PSMA PET/CT with significant PSMA avidity, defined by a SUVmax > 15 (partial volume corrected).
- Eastern Cooperative Oncology Group (ECOG): 0–1
- Patients must have a life expectancy > 6 months.
- Laboratory values:
  - White blood cells > 3.0 × 10^9^/l.
  - Platelet count > 75 × 10^9^/l.
  - Hemoglobin > 6.2 mmol/l.
  - Aspartate aminotransferase (AST) & alanine aminotransferase (ALT) < 3 x ULN.
  - Glomerular filtration rate (MDRD GFR) ≥ 50 ml/min
- Signed informed consent.

### Exclusion criteria

A potential subject who meets any of the following criteria will be excluded from participation in this study in case of:

- A known subtype other than prostate adenocarcinoma.
- Previous PSMA based radioligand treatment.
- Visceral or brain metastases.
- Any medical condition present that in the opinion of the investigator will affect patients’ clinical status when participating in this trial.
- Prior hip replacement surgery potentially influencing performance of PSMA PET/CT.
- Sjogren’s syndrome
- A second active malignancy other than prostate cancer.
- Patients who are sexually active and not willing/able to use medically acceptable forms of barrier contraception.

### Evaluation and randomization

**Fig. 2:**
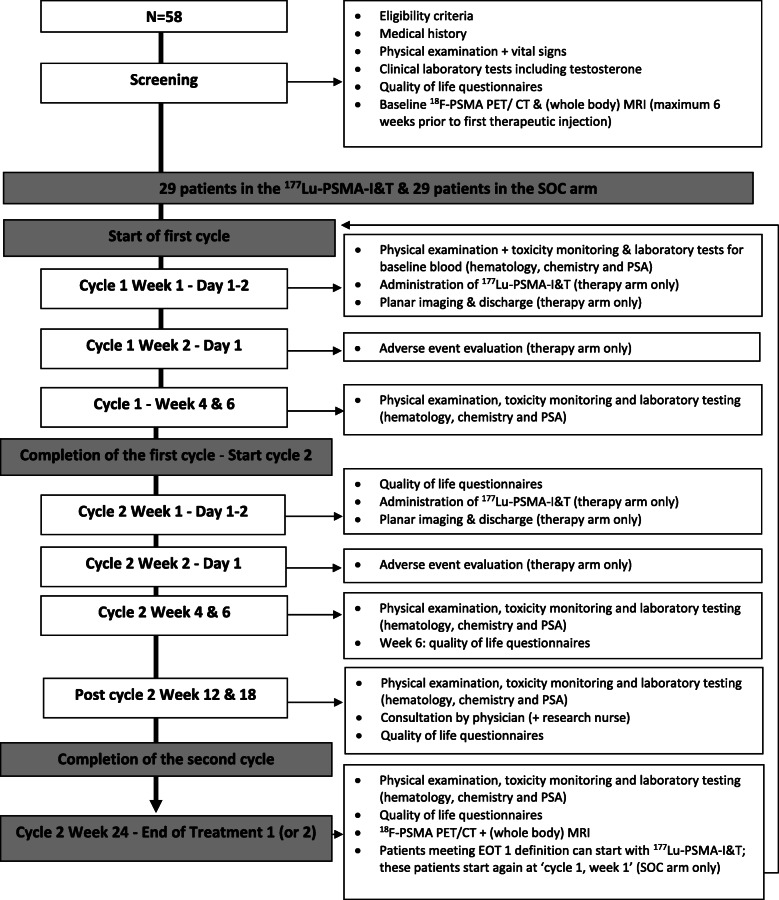
study flowchart. CT = computed tomography; EOT = end of treatment; MRI = Magnetic resonance imaging; PET = positron emission tomography; PSA = Prostate-specific antigen; PSMA = prostate specific membrane antigen; SOC = standard of care

All patients will have a screening visit that will include a blood draw to evaluate adequate organ functioning (Hb, leucocytes and white blood cell differentiation, thrombocytes, creatinine, sodium, potassium, ALT, AST, LDH, alkaline phosphatase, bilirubin, gamma-glutamyl transferase, amylase, albumin, PSA and testosterone) and quality of life questionnaires (EORTC QLQ-C30, QLQ-PR25 and the xerostomia inventory). Furthermore, ^18^F-PSMA PET and (whole body) MR imaging will be acquired to assess tumor PSMA uptake and heterogeneity. After reviewing all in- and exclusion criteria and study inclusion, patients will be randomized by a central reader in either the treatment or the SOC arm (1:1 ratio) using the randomization software of CastorEDC (https://www.castoredc.com/). See Fig. 2: study flowchart.

### Interventions

Once all screening or baseline procedures are performed, the next procedures will be followed within 6 weeks:

- Blood testing prior (< 7 days) to ‘cycle one, day one’ (for the interventional arm injection with ^177^Lu-PSMA-I&T) for baseline assessment (Hb, leucocytes and white blood cell differentiation, thrombocytes, creatinine, sodium, potassium, ALT, AST, LDH, alkaline phosphatase, bilirubin, gamma-glutamyl transferase, amylase, albumin, and PSA).
- Only for ^177^Lu-PSMA-I&T patients: the day of treatment injection (‘cycle one, day one’) pre-infusion measurement of vital signs (respiratory rate, blood pressure and heart rate). Subsequently, an intravenous (IV) dose of PSMA-I&T labeled with approximately 7.4 GBq of ^177^Lu will be slowly administered in approximately 5 min through the indwelling catheter. Following completion of the injection, a normal saline flush (approximately 10 mL) will ensure that all ^177^Lu-PSMA-I&T remaining in the infusion line is injected. The estimated radioactive dose will be determined by measuring the amount of radioactivity in the syringe pre- and post-injection, using a calibrated radioisotope dose calibrator. 30–60 min after injection vital signs (respiratory rate, heart rate and blood pressure) will be re-measured. Approximately 1–24 h after therapeutic injection, whole body imaging will be acquired using a gamma camera to exclude extravasation.

### Follow up

Once ‘cycle one, week one’ has been completed for the SOC or ^177^Lu-PSMA-I&T arm, the next procedures will be followed:

- One week after both treatment injections, patients that received ^177^Lu-PSMA-I&T will be evaluated for adverse events (by phone or physical consultation). Adverse events will be scored as defined by CTCAE v5.0.
- Both the SOC and ^177^Lu-PSMA-I&T arm, will be monitored at the outpatient clinic for adverse events, EOT 1 or 2 criteria and toxicity (including laboratory testing: Hb, leucocytes and white blood cell differentiation, thrombocytes, creatinine, sodium, potassium, ALT, AST, LDH, alkaline phosphatase, bilirubin, gamma-glutamyl transferase, amylase, albumin and PSA) every third week after ‘cycle one, day one’ (^177^Lu-PSMA-I&T application) and the week prior to the second cycle. After the second cycle, all patients will be monitored (including laboratory tests) at week 3, 6, 12 & 18.
- To evaluate quality of life, patients will be asked to fill in the EORTC-QLQ-30, EORTC-QLQ-PR 25 and the xerostomia questionnaire at the start of each (^177^Lu-PSM-I&T) cycle and 6, 12, 18 and 24 weeks after the second therapeutic injection.
- 24 weeks after the second injection, all study patients will have an end of study visit, including laboratory testing and image acquisition of ^18^F-PSMA PET and (whole body) MRI.
- In case of disease progression (defined as EOT 1 or 2), the end of study (whole body) MRI and ^18^F-PSMA PET scans should be acquired within 4 weeks and prior to the start of ADT or ^177^Lu-PSMA-I&T injection.
- SOC arm patients that have disease progression and meet EOT 1 will receive ^177^Lu-PSMA-I&T within 6 weeks of the EOT 1 visit. These patients will follow the same procedures and follow up as the interventional am patients starting ^177^Lu-PSMA. They will receive an extra (whole body) MRI and ^18^F-PSMA PET at the end of the study. If disease continue to progress and someone meets EOT 2 criteria despite of ^177^Lu-PSMA-I&T, an end study visit should also be planned within 4 weeks, including the extra (whole body) MRI and ^18^F-PSMA PET scans, and prior to the start of ADT.
- After completion of the study protocol, patients will be followed according to the standard of care.

### The labeling and purification of PSMA-I&T with ^177^Lu

^177^LuCl3 will be obtained from ITG (Garching, Germany). Good manufacturing practice (GMP)-grade PSMA-I&T will be obtained from piCHEM (Raaba-Grambach, Austria). The radiolabeling of PSMA-I&T will be performed on GRP synthesis module (Scintomics, Fürstenfeldbruck, Germany) using sterile and GMP-grade SC-105 kits. In brief, 4 mg gentisic acid and the PSMA-I&T peptide will be dissolved in 500 μL WFI and added to the reaction vessel. After addition to the ^177^LuCl3 in sodium acetate buffer and ascorbic acid the reaction will be incubated at 100 °C for 20 min. After cooling down, the product will be diluted to 16.5 ml with saline/DTPA to which 0.9 ml ethanol has been added. The radioactive solution will be filtered through a 0.22 μm filter (Millex GV. Merck, Amsterdam, The Netherlands) and dispensed into a closed glass type I container. Microbiological monitoring in class C will be performed during synthesis, filtration and dispensing. Assembling of the dispensing and filtration system will be performed in a class A isolator with a class B airlock (in a class C background). The radiolabeled PSMA-I&T will be measured for total radioactivity in an appropriately calibrated radioactive dose calibrator prior to injection.

#### Sample size calculation

After finishing the trial, the performance of the treatment is tested based on the first primary outcome; fraction of patients that have disease progression and meet the EOT 1 criteria within 6 months after cycle two. Only if the null hypothesis is rejected (hierarchical testing), the two arms can be compared for the second primary outcome; time till EOT 1 criteria.

Within the testing strategy as described, the sample size calculation needs to be performed for the first primary outcome only, with the risk of over- or underpowering the second-additional primary outcome. Therefore, an additional sample size calculation was performed for the second primary research question.

For the primary objective, the binomial test with pooled/equal variance under the null hypothesis and continuity correction was performed, with the numbers: sign level 0.05, power: 80%, fraction of treatment arm / control arm: 0.30 / 0.70 at 6 months. These assumption of the fractions in the two arms were based on the pilot study and published data [8, 20, 23, 33–35]. To obtain enough power for the test described above; 29 patients per arm are needed.

For the second primary objective, an exponential distribution for the time to meet EOT 1 criteria was assumed. The median survival time on the control treatment was 3.45 months (based on 0.70 at 6 months). If the true hazard ratio (relative risk) of control subjects relative to experimental subjects is 3.33 (computed based on the assumed fractions at 6 months), we will need to study 25 experimental subjects and 25 control subjects to be able to reject the null hypothesis that the experimental and control survival curves are equal with probability (power) 0.80. The Type I error probability associated with this test of this null hypothesis is 0.05. For a longer accrual interval (which will be the case in practice), the power will increase due to longer follow-up of individuals. That means that, in terms of power, the second test also has sufficient power.

#### Data analyses

All data is managed in CastorEDC database (https://www.castoredc.com/). After finishing the trial, the performance of the treatment is tested based on the first primary outcome. This will be tested with a binomial test with pooled/equal variance under the null hypothesis and continuity correction. Comparison between the arms is made based on the second primary outcome, by means of the log rank test, but only if the null hypothesis of the primary objective is rejected. From both study arms sample fractions with 95% confidence intervals and Kaplan Meier curves will be computed. The significance level is set at 0.05.

## Discussion

Currently, increasing data are showing that MDT for oHSPC improves PFS without significant side effects, in contrast to the toxicity related to ADT [8, 9, 11]. In this setting, ^177^Lu-PSMA is anticipated to be effective coupled with low grade toxicity. Moreover, ^177^Lu-PSMA-I&T is not limited to previous curative intended treatments like surgery or EBRT. ^177^Lu-PSMA I&T is injected intravenously and targets PSMA expressing tumors selectively. This trial will investigate if ^177^Lu-PSMA-I&T RLT is an effective treatment in oHSPC, and is currently the first study investigating ^177^Lu-PSMA-I&T in ADT-naïve setting, but also the first randomized prospective study with PSMA-I&T world-wide.

## Data Availability

The datasets used and/or analyzed during the current study are available from the corresponding author on reasonable request.

## Abbreviations

^68^Ga: Gallium-68
^18^F: Fluor-18
^177^Lu: Lutetium-177
ADT: Androgen deprivation therapy
ALT: Alanine aminotransferase
AST: Aspartate aminotransferase
CT: Computed tomography
CTCAE: Common terminology criteria for adverse events
EBRT: External beam radiotherapy
ECOG: Eastern cooperative oncology group
EOT: End of study
GMP: Good manufacturing practice
IV: Intravenous
LDH: Lactate dehydrogenase
MDRD GFR: Glomerular filtration rate
MDT: Metastases directed therapy
oHSPC: Oligometastatic hormone-sensitive prostate cancer
PC: Prostate cancer
PCWG3: Prostate cancer working group 3
PET: Positron emission tomography
PSA: Prostate-specific antigen
PSMA: Prostate-specific membrane antigen
RECIST: Response evaluation criteria in solid tumors
SOC: Standard of care
SUV: Standardized uptake value
